# The highly conserved serine threonine kinase StkP of *Streptococcus pneumoniae *contributes to penicillin susceptibility independently from genes encoding penicillin-binding proteins

**DOI:** 10.1186/1471-2180-9-121

**Published:** 2009-06-05

**Authors:** Ricardo Dias, David Félix, Manuela Caniça, Marie-Claude Trombe

**Affiliations:** 1 Antibiotic Resistance Unit, Department of Infectious Diseases, National Institute of Health Dr. Ricardo Jorge, Lisbon, Portugal; 2 INSERM Unité 858, Equipe 15, Université Paul Sabatier, Toulouse, France

## Abstract

**Background:**

The serine/threonine kinase StkP of *Streptococcus pneumoniae *is a major virulence factor in the mouse model of infection. StkP is a modular protein with a N-terminal kinase domain a C-terminal PASTA domain carrying the signature of penicillin-binding protein (PBP) and prokaryotic serine threonine kinase. In laboratory cultures, one target of StkP is the phosphoglucosamine mutase GlmM involved in the first steps of peptidoglycan biosynthesis. In order to further elucidate the importance of StkP in *S. pneumoniae*, its role in resistance to β-lactams has been assessed by mutational analysis in laboratory cultures and its genetic conservation has been investigated in isolates from infected sites (virulent), asymptomatic carriers, susceptible and non-susceptible to β-lactams.

**Results:**

Deletion replacement mutation in *stkP *conferred hypersensitivity to penicillin G and was epistatic on mutations in PBP2X, PBP2B and PBP1A from the resistant 9V clinical isolate URA1258. Genetic analysis of 55 clinical isolates identified 11 StkP alleles differing from the reference R6 allele. None relevant mutation in the kinase or the PASTA domains were found to account for susceptibility of the isolates. Rather the minimal inhibitory concentration (MIC) values of the strains appeared to be determined by their PBP alleles.

**Conclusion:**

The results of genetic dissection analysis in lab strain Cp1015 reveal that StkP is involved in the bacterial response to penicillin and is epistatic on mutations PBP 2B, 2X and 1A. However analysis of the clinical isolates did not allow us to find the StkP alleles putatively involved in determining the virulence or the resistance level of a given strain, suggesting a strong conservation of StkP in clinical isolates.

## Background

*Streptococcus pneumoniae *is one of the main aetiological agents of invasive infectious disease. Penicillin-resistant pneumococci were first observed in the 70s, and resistance to penicillin and multidrug-resistance have since then increased worldwide. Cell-wall biosynthetic enzymes named Penicillin Binding Proteins (PBP) are the targets for β-lactam antibiotics; mutations in these proteins constitute a major mechanism of resistance in clinical isolates. In laboratory strains, *murMN*, *ciaRH *and *cpoA *genes are also involved in penicillin susceptibility suggesting their involvement in cell wall metabolism [1-3]. One of the first steps of cell wall biosynthesis is catalysed by the phosphoglucosamine mutase GlmM [4]. In *Escherichia coli*, GlmM is activated by phosphorylation and it has been shown, in vitro, that GlmM of *S. pneumoniae *is a substrate for the serine/threonine kinase Stk, suggesting a role for StkP in cell wall metabolism [5,6].

StkP protein from *S. pneumoniae *contains a eukaryotic kinase domain (Hanks kinase domain) and a PASTA (penicillin-binding protein and serine threonine kinase) domain signature only found in prokaryotes and putatively involved in cell wall sensing [7]. In this study we evaluate the role of StkP in β-lactam susceptibility both in "the model laboratory strain Cp1015" and in natural clinical isolates carrying different PBP alleles.

## Methods

### Bacterial strains, plasmids and growth conditions

The plasmids and strains used in this study are described in Table 1[8]. *Escherichia coli *was grown in LB (Difco, Sparks, Maryland) supplemented or not with ampicillin (100 μg ml^-1^) (Atral, Castanheira do Ribatejo, Portugal). *S. pneumoniae *clinical isolates were grown at 35°C on Columbia agar plates supplemented with 5% horse blood (Biomerieux, Carnaxide, Portugal), in an atmosphere enriched with 5% CO_2_. Serotyping was performed by the Quellung reaction with antisera produced by the Statens Seruminstitut, Copenhagen, Denmark [9]. Laboratory unencapsulated strains derived from D39 were routinely grown in casitone and tryptone (CAT) medium at 37°C [10]. CTM transformation medium was used to induce competence and for transformation, as described previously [11]. The CSP concentration was 100 ng ml^-1 ^and DNA concentration was 1 μg ml^-1^. The chromosomal source of DNA carrying mutated PBP alleles was the 9V derivative Spain^23F^-1 clone (strain URA1258) which carries the following mutations near or within the conserved motifs on the PBPs: Gln443Glu, Thr451Ala, Glu481Gly, Ser485Ala and Thr494Ala in PBP2B, Thr338Ala, Met343Thr, Ala346Ser, Ala347Ser, Leu364Phe, Ile371Thr, Arg384Gly, Leu546Val and Asn605Thr in PBP2X, and Thr371Ala, Glu388Asp, Pro432Thr, Asn546Gly, Thr574Asn, Ser575Thr, Gln576Gly, Phe577Tyr, Leu606Ile, Asn609Asp, Leu611Phe and Thr612Leu in PBP1A. Transformants were selected on plates containing 0.1 μg ml^-1 ^and 0.5 μg ml^-1 ^penicillin, and appropriate integration of PBP mutations was confirmed by nucleotide sequencing. Plates containing 2 μg ml^-1 ^rifampicin and 10 μg ml^-1 ^chloramphenicol were used to select *rif-23 *and Δ*stkp::cat *transformants. All constructions were verified by PCR with the primers described in Table 2[6,12]. Spontaneous mutation to penicillin in DNA free medium was < 10^-9^. Penicillin G was from Atral, Castanheira do Ribatejo, Portugal, and rifampicin was from Aventis Pharma. To assess StkP and PBPs conservation 50 strains were randomly selected among those isolated between 1994 and 2005 in various areas in Portugal; they included forty invasive isolates from blood and cerebrospinal fluid and ten colonizing isolates from the nasopharynx of asymptomatic carriers. Half of the isolates (n = 25) were non-susceptible to penicillin [minimal inhibitory concentration (MIC) > 0.1 μg ml^-1^]. These isolates were compared to the following reference strains: the highly resistant serotype 9V strain URA1258, two susceptible and three non-susceptible strains provided by the ATCC and the unencapsulated strain R6 (Table 1).

**Table 1:** Strains and plasmids used in the study

Strain or plasmid	Genotype or description	**Phenotype**^a^	Source or reference
***S. pneumoniae***			
R6	Non-capsulated D39 derivative, susceptible reference strain; genome sequence available (R6)	AtbS	Laboratory stock
ATCC BAA-334	Virulent reference strain, genome sequence available (TIGR4)	AtbS	ATCC
ATCC 51916	Reference strain Tennessee 23F-4	PenR, EryR,	ATCC
ATCC 700670	Reference strain Spain 6B-2	PenR, CmR, TetR	ATCC
ATCC 700673	Reference strain Hungary 19A-6	PenR, EryR, CmR, TetR	ATCC
URA1258	Multiresistant strain closely related to Spain 23F-1 clone	PenR, CmR, TetR	[21]
Cp1015	D39 derivate, *str1*; *hexA*	SmR	[31]
Cp1016	D39 derivate, *str1*; *hexA*, *rif23*	RifR	[31]
Cp7000	Cp1015, *stkP*::*cat*	SmR CmR	This study
Pen1	Cp1015, *penA*, and *pbpX *from URA1258	SmR PenR	This study
Pen2	Cp1015, *penA*, *pbpX *and *pbp1A *from URA1258	SmR PenR	This study
Pen1STK	Cp1015Pen1, *stkP*::*cat*	SmR PenR CmR	This study
Pen2STK	Cp1015Pen2, *stkP*::*cat*	SmR PenR CmR	This study
			
***E. coli ***			
DH5α	F^-^, φ80*lac*ZΔM15, Δ(*lac*ZYA-*arg*F)U169, *deo*R, *rec*A1, *end*A1, *hsd*R17(rk^-^, mk^+^), *pho*A, *sup*E44, λ^-^, *thi*^-^1, *gyr*A96, *rel*A1	NalR	[8]
			
**plasmid**			
plSTK	3.5 kb EcoRI/SacII fragment containing *stkP *and flanking regions with *cat *cassette inserted	ApR, CmR	[6]

^a^: ApR, resistant to ampicillin; AtbS, Susceptible to all tested antibiotics; CmR, resistant to chloramphenicol; EryR, resistant to erythromycin; NalR, resistant to nalixidic acid; PenR, non-susceptible to penicillin G; RifR, resistant to rifamycin; SmR, resistant to streptomycin; TetR, resistant to tetracycline.

**Table 2:** Primers used for PCR amplification

Primer Name	Primer sequence	Gene targeted	Reference
STKP-F	5'-AGGATGCCATATGATCCAAATCGGCAA-3'	*stkP*	[6]
STKP-R	5'-TTGATTATGAATTCGCTTTTAAGGAGTAGC-3'	*stkP*	[6]
STKP-F2	5'-GTAGGACAGAATTCAAGACAAGTCTACATACA-3'	*stkP*	[6]
pbp1aF	5'-CCAGCAACAGGTGAGAGTC-3'	*pbp1A*	[12]
pbp1aR	5'-GTAAACACAAGCCAAGACAC-3'	*pbp1A*	[12]
pbp1aF2	5'-GAACTTCAAGACAAGGCAGT-3'	*pbp1A*	[12]
pbp2bF	5'-CCGTCTTAATCCCGATACC-3'	*penA*	[12]
pbp2bR	5'-ATTTTTGGGTGACTTGTTGAG-3'	*penA*	[12]
pbp2xF	5'-GGAATTGGTGTCCCGTAAGC-3'	*pbpX*	[12]
pbp2xR	5'-CATCTGCTGGCCTGTAATTTG-3'	*pbpX*	[12]

### Measurements of penicillin susceptibility

The MIC of penicillin G for the strains constructed were determined in duplicate by E-test (AB Biodisk, Solma, Sweden) according to the manufacturer's recommendations (incubation at 35°C in 5% CO_2 _for 18 to 24 H), and for clinical isolates by an agar dilution method with the testing conditions and susceptibility interpretation standards proposed by the Clinical and Laboratory Standards Institute (CLSI) [13]. Strains were considered penicillin susceptible for MIC values ≤ 0.06 μg ml^-1^, intermediate MIC for values of 0.1 – 1 μg ml^-1^, and highly resistant for MIC values ≥ 1.6 μg ml^-1^. Strains were classified as non-susceptible for MIC values ≥ 0.1 μg ml^-1^, according to CLSI criteria.

### *stkP *genotyping by amplification and nucleotide sequencing

The *stkP *gene of clinical strains was amplified by PCR using the primers listed in Table 2 and a Qiagen multiplex PCR kit (Qiagen, Hilden, Germany) according to the manufacturer's instructions. In brief, this routinely involved 40 cycles with an annealing temperature of 56°C for 1 minute. The PCR products were purified on ExoSAP-IT (USB, Cleveland, Ohio) and the nucleotide sequence was established (BigDye Cycle sequencing kit v1.1 from Applied Biosystems, Foster City, California). BioNumerics software v3.5 (Applied Maths, Sint-Martens-Latem, Belgium) was used for contig assemblages of the DNA sequences.

### Genetic diversity of the StkP kinase in 56 pneumococcal strains

The amino acid sequences deduced from the 56 *stkP *genes were aligned using the CLUSTALW program built in the MEGA version 4 software package [14]. There were a total of 637 positions in the final dataset, of which 8 were parsimony informative. The evolutionary history was inferred using the Maximum Parsimony (MP) method [15]. The bootstrap consensus tree inferred from 1000 replicates was taken to represent the evolutionary history of the *stkP *gene [16]. Branches corresponding to partitions reproduced in less than 50% of bootstrap replicates were collapsed. The MP tree was obtained using the Close-Neighbor-Interchange algorithm [17] with search level 3 [16,17] in which the initial trees were obtained with the random addition of sequences (10 replicates). The tree is drawn to scale with branch lengths calculated by the average pathway method [17] and with the number of changes over the whole sequence as units.

### Estimates of Average Evolutionary Divergence over Sequence Pairs of *stkP *within penicillin susceptibility groups

The number of amino acid and of nucleotide substitutions per site was averaged over all sequence pairs within each group by the Poisson correction method and the Maximum Composite Likelihood method, respectively, using MEGA version 4 software [14]. Standard error estimates were obtained by the bootstrap procedure (1000 replicates).

### StkP modelling

A 3D-model of the kinase domain of the StkP protein (271 residues long) of strain R6 was obtained using the sequence (accession number NP_359169). BLASTP analysis indicated that the serine-threonine kinase from strain R6 has 63% sequence identity with serine-threonine kinase of *Mycobacterium tuberculosis *(PDB ID: 1o6yA). The following structure PDB ID: 1o6yA; 1mruA.pdb, 1mruB.pdb, 1y8gB.pdb and 1zmwB.pdb were used as a template for building a homology model for the kinase domain of StkP with the SWISS-MODEL server [18,19]. Ramachandran plot analysis for phi and psi torsion angles indicated that 95.9% of residues were in the allowed region of the plot, which is more than the average cut-off of 90% used in most reliable models [20]. The final alignment adjustments and visualisation were undertaken with Deep View/Swiss-PdbViewer version 3.7.

### Genotyping of *pbp *genes

Genetic polymorphism of *penA, pbpX *and *pbp1A *genes (encoding PBP2B, PBP2X and PBP1A, respectively) of all clinical strains was investigated first by restriction fragment length polymorphism (RFLP) analysis. A number was given to each restriction pattern for each of the three *pbp *genes analysed, so the PBP profile has three numbers (for example: 4-9-7). The full genes were amplified by PCR using the primers described in Table 2 and 0.8 U of iProof Polymerase (Bio-Rad, Hercules, California) according to the manufacturer's instructions, with 35 cycles at an annealing temperature of 56°C for 30 seconds. The amplification products of *penA *and *pbpX *were digested for 1 H with 5 U of both *Hae*III and *Rsa*I restriction endonucleases. The amplification product of *pbp1A *was similarly digested with *Hae*III and *Dde*I (all restriction enzymes supplied by New England Biolabs, Beverly, Mas.). The digested products were separated on agarose gel. Dice coefficient of similarity was used for cluster analysis with the unweighted pair group method with arithmetic averages using BioNumerics software v3.5 (Applied Maths, Sint-Martens-Latem, Belgium). The position tolerance was set to 1.0% and the optimization 1.5%. For each *pbp *gene restriction pattern identified, one isolate was randomly chosen and re-amplified by PCR for nucleotide sequencing. Contig assemblages of the DNA sequencing were performed as described above.

### Nucleotide sequence accession numbers

Sequences determined in this study have been deposited in the DBJ/EMBL/GenBank database under accession numbers AM889231 to AM889284 for *stkP*, AM779386 to AM779409 for *penA*, AM779338 to AM779361 for *pbpX*, and AM779362 to AM779385 for *pbp1A*.

## Results

### Influence of *stkP *mutation on penicillin susceptibility in a model system

The role of StkP in penicillin resistance, has been assessed by genetic analysis in the laboratory transformable strain Cp1015 (Table 1). The penicillin sensitive strain Cp1015 was transformed with DNA from the serotype 9V resistant strain URA1258 related to the international multiresistant clone Spain^23F^-1 [21]. Penicillin-resistant transformants were selected on plates containing 0.1 μg ml^-1 ^of penicillin. One transformant was isolated: strain Pen1, isogenic to Cp1015 but with mutations in PBP2X and 2B and resistant up to 0.125 μg ml^-1 ^of penicillin. Strain Pen1 was then transformed with DNA from URA1258 and transformants were selected on plates containing 0.5 μg ml^-1 ^penicillin; this gave strain Pen2 isogenic to Pen1 but for mutations in *pbp1A *and resistant to 0.5 μg ml^-1 ^penicillin. Transformation of strains Cp1015, Pen1 and Pen2 with plasmid plSTK (Table 1) and selection on chloramphenicol plates gave the corresponding isogenic strains differing by their PBP and StkP alleles. The MICs of these strains were determined: the StkP^- ^allele significantly and reproducibly increased penicillin susceptibility (Table 3). The StkP^- ^mutations not only increased the penicillin susceptibility of strain Cp1015 carrying wild-type penicillin binding proteins, but was also epistatic on mutations PBP2B, 2X and 1A; therefore StkP acts upstream from the PBPs.

**Table 3:** Resistance phenotype and transformability of RX derivatives with different combinations of PBP and StkP alleles

Strain	Genotype	MIC Pen^a ^(μg ml^-1^)
URA1258	Multiresistant strain closely related to Spain 23F-1 clone	0.5–1
Cp1015	Rx derivate, *str1*; *hexA*	0.016
Cp7000	Cp1015, *stkP*::*cat*	0.008
Pen1	Cp1015, *penA *and *pbpX *from URA1258, allelic exchange mutant	0.064 – 0.125
Pen2	Cp1015, *penA*, *pbpX *and *pbp1A *from URA1258, allelic exchange mutant	0.38 – 0.5
Pen1STK	Pen1, *stkP*::*cat*	0.016 – 0.032
Pen2STK	Pen2, *stkP*::*cat*	0.032 – 0.125

^a^: MIC Pen, Minimum inhibitory concentration for penicillin. ND, not determined.

### Polymorphism of *stkP *in clinical isolates and relationship to penicillin resistance

The effect of the StkP^- ^mutation on penicillin susceptibility, as observed in an isogenic system, led us to question the importance of the *stkP *gene on penicillin susceptibility among clinical isolates. Thus, we evaluated the level of genetic conservation of the *stkP *gene and the presence of mutations related to alterations of susceptibility to penicillin on a sample of clinical isolates recovered from invasive disease and asymptomatic carriers.

**Figure 1:**
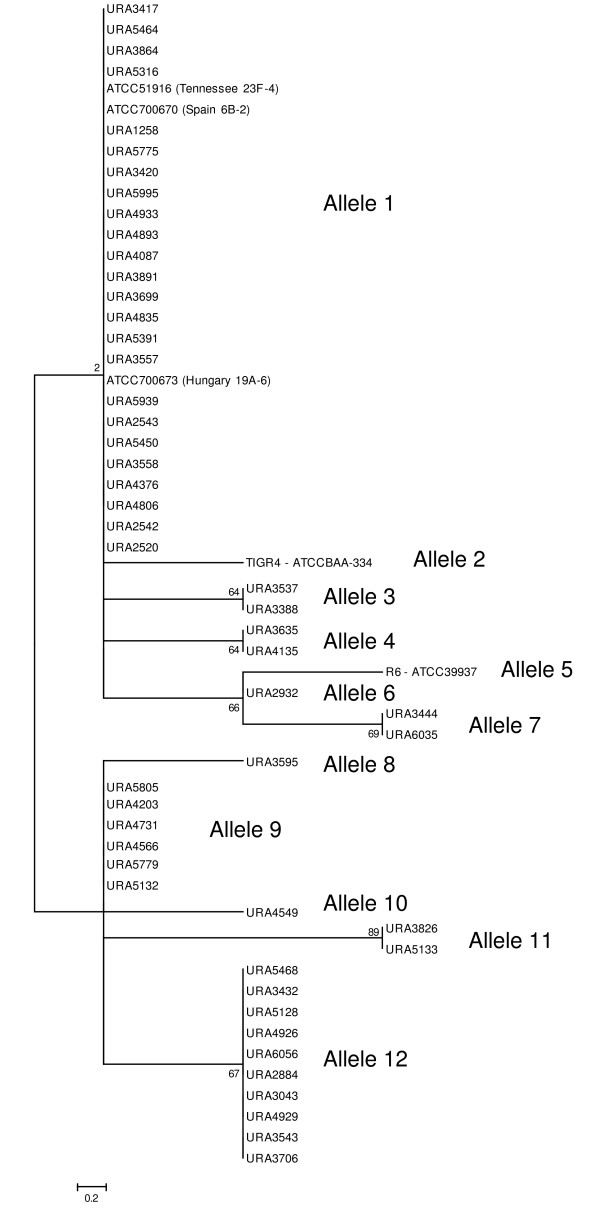
**Inference of phylogenetic history of StkP from 56 strains using the Maximum Parsimony method**. A number was given to each branch corresponding to the StkP alleles. The percentage of replicate trees in which the associated taxa clustered together in the bootstrap test (1000 replicates) are shown next to the branches.

The sequence of the *stkP *gene from 50 clinical isolates and 6 reference strains was determined. The *stkP *gene in each strain was amplified by PCR using oligonucleotides complementary to sequences at -10 and +1997 of the gene. In each case, a 2007 bp DNA fragment was obtained and the nucleotide sequences confirmed that they corresponded to *stkP*. There were 61 segregating sites (S) with a rate of segregating sites per site (pS) of 0.033, resulting in 27 allelic variants with an average of 10.26 nucleotides substitutions per sequence. Analysis of the encoded amino-acid sequences revealed 11 segregating sites (S) and a rate of segregating sites per site (pS) of 0.020, resulting in 12 allelic variants (including strain R6) with an average of 1.37 amino acid substitution per sequence (Additional file 1: Table ST1 and Figure 1). Thus, the full-size StkP protein is well conserved in invasive and colonising clinical isolates and independent of their penicillin-resistance character.

We considered PASTA domains and kinase domains individually: nucleotide divergence was higher in the 5' terminal part of the gene encoding the kinase module (*d *= 0.0072; S.E.: 0.0013) than in the 3' part of the gene encoding the PASTA modules (*d *= 0.0048; S.E.: 0.0011). By contrast, amino acid divergence was higher in the PASTA domains (*d *= 0.0037; S.E.: 0.0011) than in kinase domain (*d *= 0.0012; S.E.: 0.0007). The distribution of the amino acid allelic variants of StkP into penicillin-resistance classes was assessed (Figure 1): alleles 2, 3, 5, 6, 7, 8, 10 and 11 were found in penicillin-susceptible strains and alleles 1, 4, 9 and 12 were found both in penicillin-resistant and -sensitive strains (Additional file 1: Table ST1). The StkP amino acid sequence divergence was similar among penicillin-susceptible strains (*d *= 0.0027; S.E.: 0.0009), penicillin-intermediate strains (*d *= 0.0015; S.E.: 0.0009) and highly resistant strains (*d *= 0.0017; S.E.: 0.0011).

**Figure 2:**
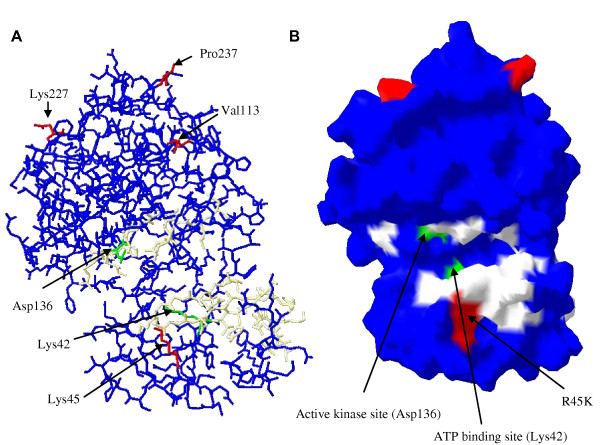
**Predicted structure of the kinase catalytic domain of StkP**. (A) Image of backbone with oxygens of the StkP kinase domain (4–274). In white, predicted catalytic zones: ATP-binding site (18–42) and kinase motif (132–144). In green: Asp136 – phosphorylation site and Lys42 – ATP binding site. The amino acid substitutions, relative to the R6 sequence, are in red: Arg45Lys, Ala113Val, Asn227Lys and Ser237Pro. (B) Image of computed molecular surface of StkP kinase domain (4–274). The colours are otherwise as in Fig. 2A.

To evaluate the effects of the StkP mutations on its kinase, a model of the enzymatic domain, amino acid 4 to 274, based on the sequence of the strain R6 was developed (Accession number: NP_359169) (Figure 2). The mutations carried by the various alleles were located outside of the catalytic site and appeared unlikely to affect the ATP binding site. Thus, these clinical isolates are unlikely to carry loss of kinase function mutations.

To evaluate the consequences of mutations in the PASTA domains on the penicillin susceptibility of clinical isolates we analysed the genetic polymorphism of PBP2B, PBP2X and PBP1A, in relation the PASTA alleles in the different isolates (Additional file 1: Table ST1). RFLP patterns 4, 5, 7, 9, 18 of PBP2B, patterns 5 to 9 of PBP2X and patterns 4 to 10, 13, 16 and 17 of PBP1A (see Additional file 1: Tables ST2, ST3 and ST4) are not associated with mutations involved in penicillin resistance, according to previous descriptions [22-30]. Four PASTA alleles (StkP alleles: 3, 7, 10 and 11) were only found in sensitive strains (URA3826, URA5133, URA3537, URA3388, URA3444, URA6035, URA4549). These strains showed PBP profiles characteristic of sensitive strains, suggesting that their MICs were determined by their PBPs rather than mutation in their PASTA sequence. The other PASTA alleles were found in all the three classes of strains (high and intermediate resistance, and susceptibility) suggesting that this allele did not affect the MIC. We checked, for each strain, that the resistance character corresponded to the PBP profile (Additional file 1: Table ST1). Findings for strain URA5132 were, however, more ambiguous: it was susceptible with a MIC of 0.006 μg ml^-1 ^despite carrying the PBP2X mutations Arg384Gly and Gln552Glu related to resistance [22]; it also carries the Val623Ala PASTA allele suggesting that it may have a putative suppressor function leading to the susceptible phenotype. However, we did not test whether the PASTA Val623Ala allele is directly involved as a suppressor of the PBP mutations, partly because mutation Val623Ala is the replacement of one non polar amino acid with another. Note that this mutation was also found in resistant (URA5805 and URA4203) and intermediate (URA4566, URA4731 and URA5779) strains and therefore it is unlikely that it determines the penicillin susceptibility of strain URA5132.

## Discussion

This work presents two different approaches for the evaluation of StkP on penicillin susceptibility. By the Cp1015 model system, we present genetic and physiological evidence of the involvement of the serine threonine kinase StkP in cell wall metabolism upstream from the steps catalysed by penicillin binding proteins PBP2B, 2X and 1A in *S. pneumoniae*. The second approach allowed us to observe that StkP is genetically conserved among clinical strains, regardless of penicillin susceptibility or site of isolation. Indeed, no change of genetic diversity or any specific amino acid substitution was found to be related to isolates recovered from invasive disease or colonizing strains. These results suggest that neither virulence nor penicillin susceptibility cause a selection pressure on StkP. However, the results obtained from the analysis of clinical strains, seem to oppose the idea of an association of StkP with virulence [31], and with penicillin susceptibility found in the model system in this work. This suggests that StkP may play an important role in the homeostasis of pneumococcus in man, regardless of both virulence and penicillin susceptibility, suggesting that none of the characteristics play a central role on StkP. In fact, it has been suggested that StkP is a global regulator of gene expression [32].

The work by Gienfing *et al*., described the conservation of StkP among clinical strains and also observed the impact of *stkP *mutation on penicillin susceptibility on a susceptible genetic background [33]. However the association between PBPs and StkP mutation were not assessed. Here, we showed that the role of StkP on penicillin susceptibility is not related to the major genetic determinants for penicillin susceptibility in pneumococci among a set of clinical and reference strains as well as in the set of penicillin resistant mutants.

A contribution of the StkP towards penicillin susceptibility, notably attributed to its PASTA domains, has already been proposed elsewhere [34], but there was previously no supporting experimental evidence. This role for StkP is consistent with previous observations showing that the phosphoglucomutase GlmM is involved in the first steps of peptidoglycan biosynthesis is a target for StkP [6]. Consistent with this notion, GlmM in *E. coli *is activated by phosphorylation [4] and in *S. aureus *functional GlmM is needed for full expression of methicillin resistance [35].

Although StkP is not essential and loss of function mutations can be obtained in laboratory conditions ([6,31] and this work), it is strongly conserved in clinical isolates, reminiscent of housekeeping genes [36]; presumably, it has an important role in natural niches. Extensive sequence analysis of StkP in susceptible and resistant pneumococcal isolates did not reveal any mutation significantly associated with susceptibility to penicillin. This suggests that *stkP *is of great importance for the cellular homeostatic mechanisms of *S. pneumoniae *and is not subject to the selective pressures caused by the β-lactams, unlike *pbp *genes presenting mosaic structures.

PASTA domains in prokaryotic serine-threonine kinases and PBP2X are involved in cell wall motif recognition [7]. Consistent with our study, Jones and Dyson reported that the PASTA domain of STK from several species showed high amino acid sequence divergence and Ka/Ks values, suggesting that PASTA domain interact with a wider range of stem-peptide ligands [7]. We report similar observations for invasive and colonizing strains. It is thus unlikely that mutation in the kinase or the PASTA domains contributes to the characteristics of the virulent strains in our collection.

Although the mechanism(s) regulating StkP activity remain(s) to be determined, it is likely that GlmM under the control of StkP is a control point for cell wall homeostasis and allows penicillin insult to the cell wall to be bypassed. Interestingly, the highly conserved serine threonine-kinase of *S. pneumoniae *is thus involved in the processes underlying three key features of bacterial physiology and evolution: virulence in animals, development of competence for genetic transformation culminating in gene transfers [7], and susceptibility to penicillin (this work). This makes StkP a potentially promising target in *S. pneumoniae *for the development of new prophylactic measurements against pneumococcal disease.

## Conclusion

In summary, the results of the present study suggest that pneumococcal serine-theonine kinase (StkP) is related to penicillin susceptibility, as demonstrated in isogenic strains. However, is a highly conserved protein, not functionally related to the major genetic determinants for penicillin susceptibility in pneumococci, being a promising target for the development of new therapies.

## Authors' contributions

RD and DF carried out the laboratory experiments. MC and MCT designed the study and RD, MC and MCT wrote the manuscript. All authors read and approved the final manuscript.

## Supplementary Material

Additional file 1 **Data Tables**. Data tables. This file contains table ST1 for the deduced amino acid substitutions in StkP and related PBP profiles of 50 clinical strains and 6 reference as well as tables ST2, ST3 and ST4 for the deduced amino acid substitutions in PBP2B; PBP2X and PBP1A, respectively, of 25 representative pneumococcal strains. Click here for file
