# Supplementary material for: The highly conserved serine threonine kinase StkP of Streptococcus pneumoniae contributes to penicillin susceptibility independently from genes encoding penicillin-binding proteins
Source: BMC Microbiol. 2009 Jun 5;9:121. doi: 10.1186/1471-2180-9-121 (PMC2700816; doi:10.1186/1471-2180-9-121)
Supplement: Additional file 1 — Data Tables. Data tables. This file contains table ST1 for the deduced amino acid substitutions in StkP and related PBP profiles of 50 clinical strains and 6 reference as well as tables ST2, ST3 and ST4 for the deduced amino acid substitutions in PBP2B; PBP2X and PBP1A, respectively, of 25 representative pneumococcal strains. [file 1471-2180-9-121-S1.pdf]

Additional Material for R. Dias *et al.*

Contents:

Table ST1 - Deduced amino acid substitutions in StkP and related PBP profiles of 50 clinical strains and 6 reference strains S-2

Table ST2 - Deduced amino acid substitutions in PBP2B in 25 pneumococcal strains S-6

Table ST3 - Deduced amino acid substitutions in PBP2X in 25 pneumococcal strains S-8

Table ST4 - Deduced amino acid substitutions in PBP1A in 25 pneumococcal strains S-10

Table ST1 - Deduced amino acid substitutions in StkP and related PBP profiles of 50 clinical strains and 6 reference strains[a]

Strains	Serotype	MIC[b] (μg/mL)	Kinase domain 45	113	227	237	Kinase[c]	TMS[d] 310	Pasta domains 385	449	453	492	500	623	PASTA[e]	Total	RFLP profile[g] PBP 2B	PBP 2X	PBP 1A	StkP allele[h]
R6		0.0125	R	A	N	S		V	A	S	A	D	V	V			4	9	7	5
URA3826	9V	0.006	.	V	K	P	3	.	E	.	.	.	.	A	2	6	4	7	10	11
URA5132	6B	0.006	.	.	K	P	2	.	.	.	.	.	.	A	1	4	9	7	4	9
URA5995	1	0.006	.	.	K	P	2	.	.	.	.	.	.	.	0	3	5	5	4	1
URA4376	6B	0.0125	.	.	K	P	2	.	.	.	.	.	.	.	0	3	9	5	13	1
URA3537	3	0.0125	.	.	K	P	2	.	.	.	S	.	.	.	1	4	5	5	9	3
URA4929	9V	0.0125	.	.	K	P	2	.	.	.	.	.	I	A	2	5	4	9	10	12
URA4933	14	0.0125	.	.	K	P	2	.	.	.	.	.	.	.	0	3	7	7	7	1
URA3388	4	0.0125	.	.	K	P	2	.	.	.	S	.	.	.	1	4	5	7	4	3
URA3444	33F	0.0125	.	.	K	.	1	.	.	.	.	A	.	.	1	3	5	8	4	7
URA3595	3	0.0125	K	.	K	P	3	.	.	.	.	.	.	A	1	5	4	5	4	8
URA3864	23A	0.0125	.	.	K	P	2	.	.	.	.	.	.	.	0	3	4	8	4	1
URA4135	15B	0.0125	.	.	K	P	2	A	.	.	.	.	.	.	0	4	4	8	4	4
URA4549	11A	0.0125	.	.	K	P	2	.	.	F	.	.	.	A	2	5	5	8	4	10
URA4893	22F	0.0125	.	.	K	P	2	.	.	.	.	.	.	.	0	3	7	5	4	1
URA5133	7F	0.0125	.	V	K	P	3	.	E	.	.	.	.	A	2	6	4	7	4	11

URA5775	23A	0.0125	.	.	K	P	2	.	.	.	.	.	.	.	0	3	4	8	4	1
URA5939	3	0.0125	.	.	K	P	2	.	.	.	.	.	.	.	0	3	4	9	6	1
URA6035	33F	0.0125	.	.	K	.	1	.	.	.	.	A	.	.	1	3	5	8	4	7
ATCC BAA334	4	0.0125	.	.	K	P	2	.	.	.	.	.	.	A	1	4	7	8	4	2
URA2543	23B	0.025	.	.	K	P	2	.	.	.	.	.	.	.	0	3	5	9	17	1
URA4087	19F	0.025	.	.	K	P	2	.	.	.	.	.	.	.	0	3	4	6	7	1
URA3417	14	0.025	.	.	K	P	2	.	.	.	.	.	.	.	0	3	7	7	7	1
URA5468	35F	0.025	.	.	K	P	2	.	.	.	.	.	I	A	2	5	4	9	4	12
URA2542	22F	0.05	.	.	K	P	2	.	.	.	.	.	.	.	0	3	18	7	4	1
URA2520	6	0.05	.	.	K	P	2	.	.	.	.	.	.	.	0	3	9	8	4	1
URA2932	19F	0.05	.	.	K	.	1	.	.	.	.	.	.	.	0	2	4	6	7	6
URA4566	6B	0.1	.	.	K	P	2	.	.	.	.	.	.	A	1	4	6	3	8	9
URA4731	14	0.1	.	.	K	P	2	.	.	.	.	.	.	A	1	4	3	18	4	9
URA5391	23F	0.1	.	.	K	P	2	.	.	.	.	.	.	.	0	3	1	1	5	1
ATCC 51916	23F	0.1	.	.	K	P	2	.	.	.	.	.	.	.	0	3	8	12	12	1
URA5779	15A	0.1	.	.	K	P	2	.	.	.	.	.	.	A	1	4	13	11	6	9
URA3635	19A	0.1	.	.	K	P	2	A	.	.	.	.	.	.	0	4	11	14	5	4
URA3557	19C	0.1	.	.	K	P	2	.	.	.	.	.	.	.	0	3	1	1	4	1
URA3891	23F	0.1	.	.	K	P	2	.	.	.	.	.	.	.	0	3	1	1	7	1
URA3558	23F	0.2	.	.	K	P	2	.	.	.	.	.	.	.	0	3	5	16	16	1

URA3699	19F	0.5	.	.	K	P	2	.	.	.	.	.	.	.	0	3	2	2	18	1
URA5450	24F	0.5	.	.	K	P	2	.	.	.	.	.	.	.	0	3	15	8	11	1
URA5128	14	0.5	.	.	K	P	2	.	.	.	.	.	I	A	2	5	10	13	2	12
URA4806	19A	0.5	.	.	K	P	2	.	.	.	.	.	.	.	0	3	15	13	11	1
URA4835	19A	0.5	.	.	K	P	2	.	.	.	.	.	.	.	0	3	15	13	11	1
URA4926	9V	0.5	.	.	K	P	2	.	.	.	.	.	I	A	2	5	10	13	1	12
URA3432	9V	0.8	.	.	K	P	2	.	.	.	.	.	I	A	2	5	10	13	1	12
URA3706	9V	0.8	.	.	K	P	2	.	.	.	.	.	I	A	2	5	10	13	1	12
URA5464	19A	0.8	.	.	K	P	2	.	.	.	.	.	.	.	0	3	15	13	11	1
URA3420	19A	1	.	.	K	P	2	.	.	.	.	.	.	.	0	3	15	13	11	1
URA5805	19A	1.6	.	.	K	P	2	.	.	.	.	.	.	A	1	4	17	19	14	9
URA4203	14	1.6	.	.	K	P	2	.	.	.	.	.	.	A	1	4	10	13	3	9
URA1258	9V	1.6	.	.	K	P	2	.	.	.	.	.	.	.	0	3	10	13	1	1
URA2884	14	1.6	.	.	K	P	2	.	.	.	.	.	I	A	2	5	10	13	1	12
URA5316	19C	1.6	.	.	K	P	2	.	.	.	.	.	.	.	0	3	10	13	1	1
ATCC 700673	19A	2	.	.	K	P	2	.	.	.	.	.	.	.	0	3	14	4	15	1
URA3043	14	2	.	.	K	P	2	.	.	.	.	.	I	A	2	5	10	10	1	12
URA3543	14	2	.	.	K	P	2	.	.	.	.	.	I	A	2	5	16	15	1	12
ATCC 700670	6B	2	.	.	K	P	2	.	.	.	.	.	.	.	0	3	19	17	1	1
URA6056	14	3.2	.	.	K	P	2	.	.	.	.	.	I	A	2	5	12	13	1	12

[a]: The amino acid positions in strain R6 were used as reference. All found amino acid substitutions are shown.

[b]: MIC, Minimum Inhibitory Concentration of penicillin.

[c]: Kinase, total number of amino acid substitutions in kinase domain of StkP (12-273).

[d]: TMS, transmembrane segment

[e]: PASTA, total number of amino acid substitutions in PASTA domain of StkP (366-651).

[f]: Total number of amino acid substitutions in StkP.

[g]: RFLP profile, PBP restriction fragment length polymorphism pattern for PBP2B – PBP2X – PBP1A.

[h]: Numbers correspond to each genetic lineage obtained from the Maximum Parsimony analysis (according to Fig. 1).

Table ST2 - Deduced amino acid substitutions in PBP2B in 25 pneumococcal strains

			PBP 2B[a]														
PBP 2B pattern[b]	Number of strains	MIC[c]	443 Q	451 T	481 E	485 S	488 G	494 T	614 L	624 A	629 A	630 D	633 Q	635 T	DM[d]	TP[e]	ORF[f]
4, 5, 7, 9	8	0.0125 - 0.2	.	.	.	.	.	.	.	.	.	.	.	.	0 -3	0 -1	0 -4
6	1	0.1	.	A	G	.	.	S	.	.	.	G	E	N	0	13	18
18	1	0.05	.	A	G	.	.	S	S	.	E	G	E	N	1	12	17
11	1	0.1	.	A	G	.	.	S	.	.	.	.	.	.	6	6	13
3	1	0.1	E	A	G	.	A	A	A	.	E	G	E	N	1	32	37
1	1	0.1	E	A	G	.	A	A	S	.	E	G	E	N	1	34	40
13	1	0.1	.	A	G	.	.	S	A	.	E	G	E	N	1	10	16
19	1	2	E	A	G	.	.	S	A	.	.	G	E	N	2	11	14
14	1	2	.	A	G	.	.	S	.	.	.	.	.	.	2	15	18
17	1	1.6	E	A	G	A	.	A	T	G	.	G	E	N	9	31	50
8	1	0.1	.	.	.	.	.	.	.	G	.	G	E	N	2	3	14
16	1	2	E	A	G	A	.	A	T	G	.	G	E	N	5	21	37
12	1	3.2	E	A	G	.	.	S	T	G	.	G	E	N	6	33	50
10	3	0.5-2	E	A	G	A	.	A	.	.	.	.	.	.	7	12	20

2	1	0.5	E	.	G	.	A	A	A	.	.	G	K	N	37	33	83
15	1	0.5	.	A	G	.	.	S	.	.	.	.	.	.	4	12	17

[a]**:** The amino acid positions of strain R6 were used as reference. The positions within or close to conserved motifs in the PBP2B are shown.

[b]**:** PBP2B pattern, Restriction fragment length polymorphism pattern for PBP2B.

[c]**:** MIC, Minimum Inhibitory Concentration of penicillin (μg/mL).

[d]**:** DM, number of amino acid substitutions in the dimer domain.

[e]**:** TP, number of amino acid substitutions in the transpeptidase domain.

[f]**:** ORF, number of amino acid substitutions in the open reading frame.

Table ST3 - Deduced amino acid substitutions in PBP2X in 25 pneumococcal strains

			PBP 2X[a]																			
PBP 2X pattern[b]	Number of strains	MIC[c]	338 T	339 M	343 M	346 A	347 A	364 L	369 A	371 I	384 R	394 H	400 M	546 L	550 T	552 Q	595 Y	605 N	DM[d]	TP[e]	PASTA[f]	ORF[g]
5, 6, 7, 8, 9	5	0.0125-0.025	.	.	.	.	.	.	.	.	.	.	.	.	.	.	.	.	0	1-2	0	1-2
7	1	0.0125	.	.	T	.	.	.	.	.	.	.	.	.	.	.	.	.	0	1	0	2
16	1	0.2	.	.	T	.	.	.	V	.	.	L	.	.	.	.	.	.	16	12	3	37
6	3	0.025-0.5	.	.	.	.	.	.	.	.	G	.	.	.	.	E	.	.	0-2	13-24	3-5	25-44
3	1	0.1	A	.	T	.	.	.	V	.	G	.	.	.	.	.	.	.	3	8	1	17
14	1	0.1	A	.	T	.	S	.	V	.	G	.	.	.	.	.	.	.	1	9	4	19
7	1	0.05	.	.	T	.	.	.	.	.	G	.	.	.	.	E	.	.	0	10	0	10
1	1	0.1	.	.	T	.	.	.	.	.	G	.	.	.	.	E	.	.	4	23	15	54
11	1	0.1	.	.	.	.	.	.	.	.	.	.	.	.	.	E	.	.	1	21	2	27
4	1	2	A	.	T	S	S	S	V	T	G	.	.	.	.	.	.	.	3	28	20	65
2	1	0.5	A	.	T	S	S	F	.	T	G	.	.	.	.	.	.	.	18	32	20	104
17	6	0.5 -2	A	.	T	S	S	F	.	T	G	.	.	V	.	.	.	T	2-8	34-37	1-21	45-79
12	1	0.1	A	F	.	S	S	F	.	T	G	.	T	V	A	.	.	T	2	37	21	74
13	1	3.2	A	F	.	S	S	F	V	T	G	.	T	V	.	.	F	T	3	42	23	82

[a]: The amino acid positions of strain R6 were used as reference. The positions within or close to conserved motifs in the PBP2X are shown.

[b]: PBP 2X pattern, Restriction fragment length polymorphism pattern for PBP2X.

[c]: MIC, Minimum Inhibitory Concentration of penicillin (µg/mL).

[d]: DM, number of amino acid substitutions in the dimer domain.

[e]: TP, number of amino acid substitutions in the transpeptidase domain.

[f]: PASTA, number of amino acid substitutions in PASTA – Penicillin-binding protein and serine/threonine kinase associated domain.

[g]: ORF, number of amino acid substitutions in the open reading frame.

Table ST4 - Deduced amino acid substitutions in PBP1A in 25 pneumococcal strains

PBP 1A pattern[b]	Number of strains	MIC[c]	PBP 1A[a]														
			371 T	388 E	432 P	546 N	574 T	575 S	576 Q	577 F	606 L	609 N	611 L	612 T	TG[d]	TP[e]	ORF[f]
4, 5, 6, 7, 8, 9, 10, 13, 16, 17	13	0.0125 - 0.2	.	D	.	.	.	.	.	.	.	.	.	.	1-4	1-28	7-56
4	1	0.1	.	D	.	.	N	T	G	Y	.	D	.	.	1	11	19
15	1	2	.	D	.	G	N	T	G	Y	V	D	.	.	13	44	90
1, 2, 3	6	0.5 - 3.2	A	D	T	G	N	T	G	Y	I	D	F	L	2	36-44	43-60
11, 18	2	0.5	A	D	T	G	N	T	G	Y	I	D	F	Y	1	42	70
12, 14	2	0.1-1.6	S	D	T	G	N	T	G	Y	I	D	F	L	1-12	43-47	60-80

[a]: The amino acid positions of strain R6 were used as reference. The positions within or close to conserved motifs in the PBP1A are shown.

[b]: MIC, Minimum Inhibitory Concentration of penicillin (μg/mL).

[c]: PBP 1A pattern, restriction fragment length polymorphism pattern of PBP1A.

[d]: TG, number of amino acid substitutions in the transglycosylase domain.

[e]: TP, number of amino acid substitutions in the transpeptidase domain.

[f]: ORF, number of amino acid substitutions in the open reading frame.
